# Broad-spectrum antiviral agents

**DOI:** 10.3389/fmicb.2015.00517

**Published:** 2015-05-22

**Authors:** Jun-Da Zhu, Wen Meng, Xiao-Jia Wang, Hwa-Chain R. Wang

**Affiliations:** ^1^Key Laboratory of Zoonosis of Ministry of Agriculture, College of Veterinary Medicine, China Agricultural UniversityBeijing, China; ^2^Department of Biomedical and Diagnostic Sciences, College of Veterinary Medicine, The University of Tennessee, KnoxvilleTN, USA

**Keywords:** broad-spectrum, antiviral agent, viral entry, replication, cellular defense

## Abstract

Development of highly effective, broad-spectrum antiviral agents is the major objective shared by the fields of virology and pharmaceutics. Antiviral drug development has focused on targeting viral entry and replication, as well as modulating cellular defense system. High throughput screening of molecules, genetic engineering of peptides, and functional screening of agents have identified promising candidates for development of optimal broad-spectrum antiviral agents to intervene in viral infection and control viral epidemics. This review discusses current knowledge, prospective applications, opportunities, and challenges in the development of broad-spectrum antiviral agents.

The epidemic viral diseases of severe acute respiratory syndrome (SARS), Middle East respiratory syndrome (MERS), Western/Eastern equine encephalitis (WEE/EEE), and Ebola, as well as pandemic influenza A (H1N1) occurred in 2003, 2012, 2013, 2014, and 2009, respectively. The emerging SARS, H1N1, and MERS, as well as re-emerging WEE/EEE and EBOVes are lethal and transmissible through travelers. They have been identified as high priority biodefense pathogens by the United States. The high virulence of these viruses and the absence of effective therapies have posed an ongoing threat to the public health. The conventional one-bug-one-drug paradigm is insufficient to address the challenge of emerging and re-emerging viral pathogens, and few drugs are currently available to a prompt control of epidemic viral diseases (Chan et al., 2013; Raveh et al., 2013; Carossino et al., 2014). Thus, it is imperative to develop a broad-spectrum class of antiviral agents. Current strategies for the development of broad-spectrum antiviral agents focus on two aspects of targeting viral infectivity and modulating host defense system, as discussed in this review.

## Agents Target on Viral Infectivity

**FIGURE 1:**
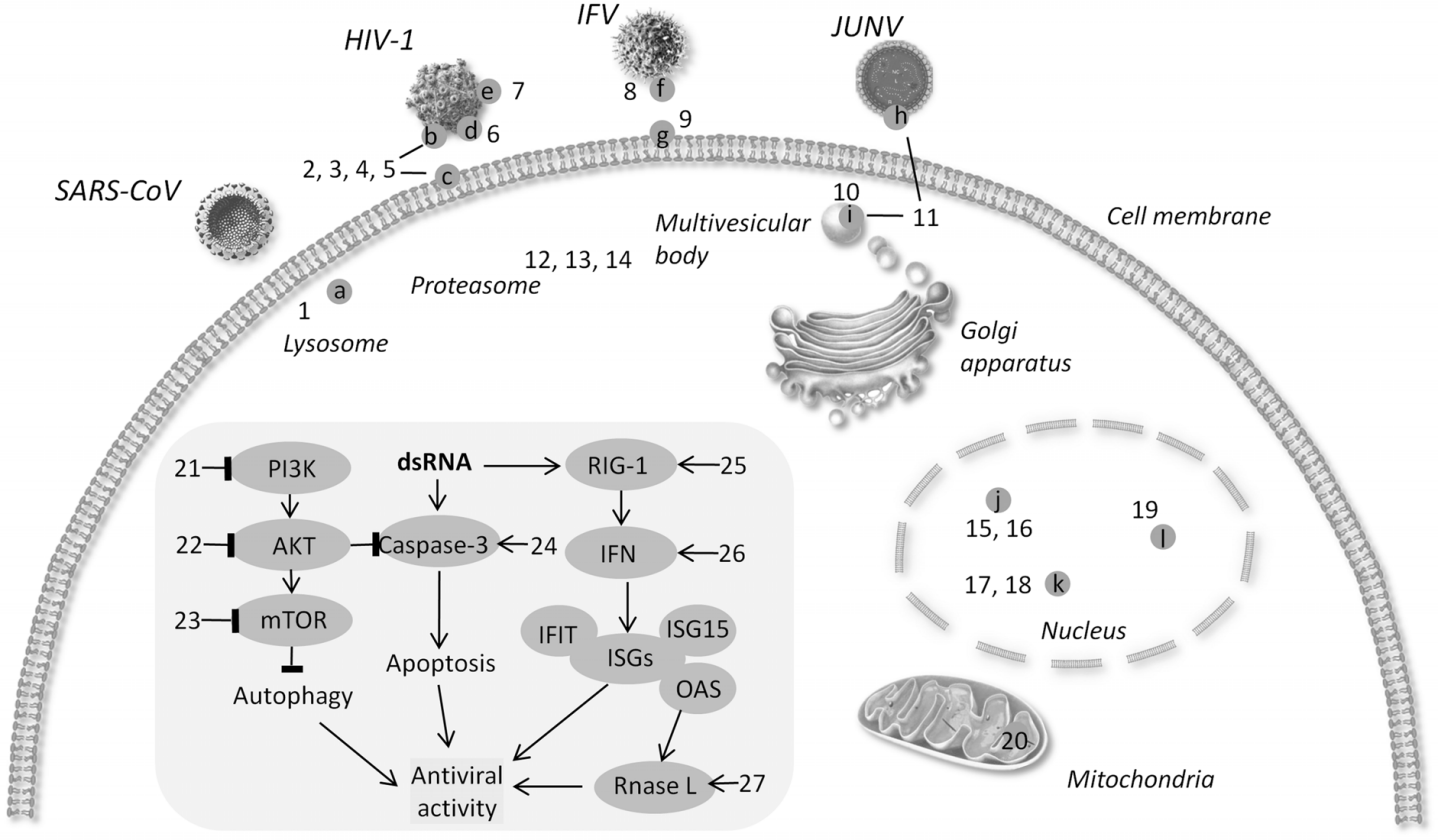
**Schematic diagram of targets for broad-spectrum antiviral agents.** (a) Cathepsin L (b) gP120 (c) CD4 (d) six-helix bundle (e). N-glycan terminal mannose residues (f) hemagglutinin (g) sialic acid (h) protein Z (i) TSG101 (j) viral RNA polymerase (k) DHODH l. XPO1 → activate - l block. (1) 5705213 (2) PVP-coated nano-silver (3) HP-OVA (4) CV-N 5. eCD4Ig. (6) p20 (7) Griffithsin (8) CR6261 (9) DAS181 (10) FGI-104 (11) Compound 0013 (12) VL-01 (13) Bortezomib (14) MG132 (15) JMN3-003 (16) BCX4430. (17) Compound A3 (18) Cmp1 (19) KPT-335 (20) Antimycin A (21) BEZ-235. (22) Akt-IV (23) Rapamycin (24) DRACOs (25) 5′pppRNA (26) GSK983. (27) Compounds C1 and C2.

Current strategies of controlling viral infectivity focus on identification of agents capable of intervening in the essential steps for viral infection, including viral attachment, fusion/endocytosis, replication, assembly and budding in addition to drugs targeting viral envelope, as detailed in the following subsections, and in **Table 1** and **Figure 1**.

**Table 1:** The antivirals target on viral infectivity.

Name	Mechanism/Target position	Derivation	Target viruses	Experiment	Reference
CR6261	Act on the hemagglutinin	Monoclonal antibody	A serotypes of IFV-A	*In vivo*	Friesen et al. (2010)
DAS181	Hydrolyze cell surface receptor	Sialidase fusion protein	IFV-A and PIV	*In vivo*	Hedlund et al. (2010)
PVP-coated nanosilver	Inhibit gp120 binding receptors	Nano-silver modification	HIV-1, etc.	*In vitro*	Lara et al. (2010)
HP-OVA	Block glycoprotein- binding receptors	Egg white protein modification	HIV-1, etc	*In vitro*	Li et al. (2010)
GRFT	Block glycoprotein- binding receptors	Plant extract	HIV-1, etc	*In vivo*	Barton et al. (2014)
CV-N	Block gp120 to mediate fusion	Extract from cyanobacterium	HIV and EBOV, etc.	*In vitro*	Botos and Wlodawer (2003)
eCD4-Ig	Inactivate gp120	Recombinant	HIV and SIV	*In vitro*	Gardner et al. (2015)
5705213	Block cathepsin cleavage	Synthesis	Broad-spectrum, e.g., SARS-CoV	*In vitro*	Elshabrawy et al. (2014)
P20	Block six-helix bundle formation	Synthesis	Various HIV-1 strains	*In vitro*	Zhu et al. (2010)
C-AU	Block six-helix bundle formation	Recombinant protein	NDV and IBV	*In vivo*	Wang et al. (2011)
BCX4430	Inhibit viral RNA polymerase	Synthesis	RNA viruses, e.g., YFV	*In vivo*	Warren et al. (2014)
JMN3-003	Inhibit viral RNA polymerase	Synthesis	Ortho- and para-myxoviruses	*In vitro*	Krumm et al. (2011)
T705	Target viral RNA polymerase	Synthesis	RNA viruses, e.g., EBOV	*In vivo*	De Clercq (2015)
A3	Interrupt biosymthesis of pyrimidine	Synthesis	Broad-spectrum, e.g., IFV-A and B	*In vitro*	Hoffmann et al. (2011)
Ribavirin	Inhibit synthesis of viral RNA	Synthesis	RSV and HCV, etc	*In vivo*	Naik and Tyagi (2012) (Review)
Cmp1	Anti-DHODH activity	Synthesis	broad-spectrum, e.g., HCMV	*In vivo*	Marschall et al. (2013)
antimycin A	Inhibit the cellular mitochondrial electron transport chain	Extract from *Streptomyces*	RNA viruses, e.g., EEV	*In vivo*	Raveh et al. (2013)
Nitazoxanide	Disrupt viral protein glycosylation	Synthesis	Broad-spectrum, e.g., HIV and DENV	*In vivo*	Rossignol (2014) (Review)
GC373/375/376	Target 3C and 3CL proteases	Synthesis	Positive-sense RNA viruses, e.g., SARS-CoV	*In vitro*	Kim et al. (2012)
KPT-335	Nuclear export inhibitor	Synthesis	A serotypes of IFV-A	*In vivo*	Perwitasari et al. (2014)
FGI-104	Block hijacking TSG101	Synthesis	Broad-spectrum, e.g., EBOV	*In vivo*	Kinch et al. (2009)
0013	Block viral protein interact with cellular TSG101	Synthesis	RNA viruses, e.g., JUNV	*In vitro*	Lu et al. (2014)
LJ001	Interrupt viral envelope	Synthesis	Enveloped viruses	*In vitro*	Wolf et al. (2010)
MP7-NH2	Inactivate virus	Biosynthesis	Enveloped viruses	*In vitro*	Sample et al. (2013)
Arbidol	Block virus interact with cell membrane	Synthesis	Enveloped viruses, e.g., IFV-A	*In vivo*	Blaising et al. (2014) (Review)

### Viral Attachment

Viral attachment to host cells is a specific interaction between viral surface proteins and host cell surface receptors and is a critical step for most viral entry into cells. Thus, blocking the interaction between viral particles and host cells is an important task being seriously considered in development of broad-spectrum antiviral agents to control viral infection, such as IFV and HIV-1.

IFV-A infects host cells through interaction of its surface antigen haemagglutinin with host cell surface receptor complex containing sialic acid (Nicholls et al., 2008). The highly conserved epitope at membrane-proximal stem of hemagglutinin has been successfully targeted by the specific monoclonal antibody CR6261 to inhibit IFV-A infection via blocking the conformational changes of haemagglutinins during viral attachment to host cells. Pre- or post-treatment of mice with CR6261 effectively inhibit infection with various serotypes of IFV and protect animals from IFV infection, demonstrating CR6261′s potential for a broad-spectrum antiviral agent (Friesen et al., 2010). On the other hand, the sialic acid of host cell surface receptor is also a recognized target for development of broad-spectrum antiviral agents. Sialidase hydrolyzes cell surface sialic acid and prevents viral attachment to cells. DAS181, a sialidase fusion protein consisting of a sialidase enzymatic domain and an amphiregulin glycosaminoglycan binding sequence to respiratory epithelium, has been shown to efficiently protect mice from infection of various serotypes of IFV, such as A/PR/8/34 (H1N1) and A/Victoria/3/75 (H3N2), as well as PIV. Treatment of IFV-infected animals with DAS181 also prevents pneumonia, indicating that DAS181 treatment prevents animals from secondary bacterial infection (Hedlund et al., 2010). Thus, CR6261 and DAS181 are candidates for developing broad-spectrum antiviral agents to control infection by IFV variants.

The HIV-1 particle carries only one envelope glycoprotein precursor gp160, which can be cleaved by host proteases, such as Furin, into gp120 and gp41; the resulting gp120 binds to the cell surface receptor CD4 and coreceptors (CCR5, CXCR4) for viral attachment and entry into cells (Wilen et al., 2012). Three reagents silver, ovalbumin (OVA), and lectin have been considered for development of broad-spectrum antiviral agents; they are all cost-efficient materials for antiviral agent development. Silver is a common expedient for cooking procedures and preserving water from contamination. Coating nano-silver with polyvinylpyrrolidone (PVP) has been shown to competitively bind to gp120, resulting in blocking viral attachment to the host cell receptor CD4 and preventing viral infection (Galdiero et al., 2011). In addition, PVP-coated nano-silver exhibits antiviral ability to directly inhibit the infectivity of viral particles in a cell-independent manner (Lara et al., 2010). However, what other metal nanoparticles may interfere with the infectivity of viral particles for developing broad-spectrum antiviral agents remains to be investigated. Similarly, OVA shows anti-viral ability, and 3-hydroxy phthalic anhydride-modified OVA (HP-OVA) exhibits a broad-spectrum antiviral activity by interfering with gp120 binding to CD4 (Li et al., 2010). Many natural lectins, such as Concanavalin A and Lentil lectin isolated from plants, have been shown to be cytotoxic, to non-specifically activate T cells, and to agglutinate red blood cells; these concerns have limited the use of lectins in antiviral drug development (Barton et al., 2014). Mori’s group revealed that the 12 kDa red algae lectin Griffithsin (GRFT) binds *N*-glycan terminal mannose residues of gp120, blocks viral binding to CD4-expressing cells, and inhibits HIV-1 entry into cells in a glycosylation-dependent manner (Mori et al., 2005). GRFT has been shown to possess a broad-spectrum antiviral activity, and it is potentially safe in systemic treatment of HIV-1 infection without inducing pro-inflammatory cytokines (Mori et al., 2005; Barton et al., 2014). Cyanovirin-N (CV-N), a natural lectin isolated from the *cyanobacterium Nostoc ellipsosporum*, has been shown its high-affinity to interact with the *N*-linked high-mannose oligosaccharides of HIV gp120 and block the ability of gp120 to mediate membrane fusion (Botos and Wlodawer, 2003). However, the associated cytotoxicity and immunogenicity have hindered the development of CV-N to be a viable therapeutic agent. CV-N also inhibits EOBV and HCV infection effectively by interacting with viral envelope glycoproteins at the early stages of viral entry (Barrientos et al., 2004; Helle et al., 2006). Recently, (GGGGS)3, which is a flexible and hydrophilic linker, was used to conjugate with CV-N to produce LCV-N; then PEG was used to modify the N-terminus of LCV-N to produce PEGylated LCV-N (PEG-LCV-N); and PEG-LCV-N is effective to control IFN infection *in vivo* (Wu et al., 2015). Accordingly, CV-N and its derivatives, such as PEG-LCV-N, should be further developed and used as broad-spectrum antiviral agents. Thus, PVP-coated nano-silver particles, HP-OVA, GRFT, and CV-N should be further studied for developing broad-spectrum antiviral agents to control HIV-1 infection.

Broad-spectrum neutralizing antibodies (bNAbs) are being developed to target HIV gp120 for inhibition of HIV infection. However, all the tested bNAbs were able to neutralize 10–50% of HIV-1 isolates (Su and Moog, 2014). Recent development of antiviral agents recognizes that two most conserved epitopes of HIV-1 gp120 are CD4- and CCR5-binding sites (Gardner et al., 2015). Fusion of CD4-Ig, which is a recombinant fusion protein consisting of CD4 and Fc region, with a small CCR5-mimetic sulfopeptide resulted in an effective gp120-targeted agent eCD4-Ig. eCD4-Ig has been shown to effectively neutralize 100% of a panel of HIV-1, HIV-2, and simian immunodeficiency viruses, including bNAbs-resistant viral isolates (Gardner et al., 2015). Accordingly, eCD4-Ig should be further developed as an antiviral agent to control HIV infection.

### Membrane Fusion and Endocytosis

Severe acute respiratory syndrome coronavirus, EBOV, HeV, and NiV are highly pathogenic to a broad range of hosts including human and various species of animals. The entry of these viruses into host cells is dependent on cathepsin L (CatL), which is a cellular lysosomal protease required for processing gS-glycoprotein cleavage, to generate fusion of viral envelope with cellular membrane after viral attachment (Pager and Dutch, 2005; Simmons et al., 2005; Pager et al., 2006; Kaletsky et al., 2007). High throughput approaches have identified 50 molecules from 5000-molecule libraries by their ability to inhibit CatL and block viral entry into cells. The small molecule 5705213 and its derivative 7402683 have been shown to efficiently inhibit CatL-mediated cleavage of gS-glycoprotein and effectively block SARS-CoV entry into cells (Elshabrawy et al., 2014). The molecule 5705213 also blocks the entry of EBOV, HeV, and NiV into cells via inhibition of glycoprotein cleavage (Elshabrawy et al., 2014). Accordingly, identification of small molecules to inhibit CatL is a promising approach of developing broad-spectrum antiviral agents to interfere with virus-cell membrane fusion, endocytosis, and viral entry into cells.

Binding the gp120 of HIV-1 to cellular receptors triggers a conformational change of the subunit gp41 for the fusion of viral envelope and cellular membrane (Wilen et al., 2012). The gp41 is a transmembrane protein, and the core of gp41 consists of three units of heptad repeat 1 (HR1) and three units of HR2; HR2 units surround the HR1 units in an anti-parallel manner to form a six-helix bundle structure. Conformational changes of the six-helix bundle activate its function to mediate the fusion of viral and cellular membranes together (Colman and Lawrence, 2003). The HR2-mimicking peptide Enfuvirtide (Fuzeon, a currently, clinically approved peptide fusion inhibitor) can bind HR1 and interfere with conformational changes, thereby blocking the formation of active six-helix bundle structure (Eckert and Kim, 2001). Although Enfuvirtide is effective in control of wild-type HIV-1 and variants, Enfuvirtide treatment results in resistant HIV-1 variants, which are also resistant to reverse transcriptase inhibitors (such as Zidovudine) and protease inhibitors (such as Ritonavir; Eckert and Kim, 2001). Thus, development of next-generation inhibitors of HRs is urgent. Recently, the P20, homologous to human troponin I type 3 interacting kinase (TNNI3K)-like protein, is identified by screening a DNA library of human bone marrow cells through yeast two-hybridization method (Zhu et al., 2010). The P20 does not share any homology with Enfuvirtide, but it binds to six-helix bundle and inhibit infection by various HIV-1 strains. It is reported that P20 variants with a common motif (WGRLEGRRT) exhibit potent activity to inhibit HIV-1 infection (Zhu et al., 2010); apparently, this common motif can be used as a lead to develop optimal inhibitors for controlling HIV-1 infection.

Similar to HIV-1, a six-helix bundle structure is reported to mediate the entry of class I enveloped viruses into host cells, including coronavirus and paramyxovirus (Liu et al., 2004; Wang et al., 2005); the six-helix bundle structure appears to be an optimal target for developing broad-spectrum antiviral agents. To achieve this end, the HR2 sequences of the paramyxovirus NDV and the coronavirus IBV were systemically analyzed. The primary structure of each helix consists of heptad repeats containing seven commonly residues (*a–b–c–d–e–f–g*)_n_. Replacement of the non-conservative sites *b, c, f*, and *g* with the charged residues glutamic acid or lysine followed by screening revealed a novel peptide AU (VNKK**I**EE**ID**KK**I**EE**LN**KK**L**EE**LE**KK**L**EE**VN**KK) that is able to completely inhibit single or double infection of cells with NDV and IBV *in vitro* and *in vivo* (Wang et al., 2011). Furthermore, tagging a cholesterol unit to the N-terminus of AU, resulting in C-AU, not only prolongs the half-life of AU *in vivo* but also increases its potency to control viral infection. For example, intramuscular injection of chicken with C-AU one day prior to virus inoculation and then every 3 days for three times after viral inoculation resulted in a 70% protection of animals from NDV infection (Li et al., 2013). This *in vivo* study demonstrated feasibility of using antiviral peptides in clinical application to control viral infection.

### Viral Replication

RNA polymerase is essential for the replication of numerous RNA viruses (te Velthuis, 2014). BCX4430, a novel nucleoside analog, is an inhibitor specific for viral RNA polymerase and exhibits broad-spectrum antiviral activity against viruses including filoviruses, flaviviruses, arenaviruses, paramyxoviruses, coronaviruses, and bunyaviruses (Warren et al., 2014). BCX4430 treatment completely protected golden hamsters from death caused by infection with YFV (flavivirus), and the treatment also improved symptoms including weight loss and viremia. BCX4430 also completely protected cynomolgus macaques from infection with Marburg virus, a hemorrhagic fever virus of the *Filoviridae* family, even it administered 48 h after viral infection (Julander et al., 2014; Warren et al., 2014). Similarly, JMN3-003 can block the replication of various viruses (paramyxoviruses, orthomyxoviruses, etc.) via specific inhibition of viral RNA polymerases; interestingly, JMN3-003 treatment does not affect mRNA synthesis or protein translation of host cells (Krumm et al., 2011). Both BCX4430 and JMN3-003 are able to specifically target viral RNA polymerases without causing side effects to hosts. In addition, resistant viral variants were not detectable in animals after treatment with BCX4430 or JMN3-003 (Krumm et al., 2011; Warren et al., 2014). The pyrazine derivative favipiravir (T-705) targets viral RNA-dependent RNA polymerases, and T705 treatment inhibits almost all RNA viruses without affecting DNA or RNA synthesis of host cells. Clinically, T-705 was reportedly effective in controlling EBOV infection (Oestereich et al., 2014; De Clercq, 2015). In addition, T-705 treatment of IFV-A-infected cells did not result in detectable resistant variants (Mendenhall et al., 2011). Accordingly, agents, targeting viral RNA polymerases, are optimal candidates of broad-spectrum antiviral agents to be fully developed for the control of RNA viruses.

Dihydroorotate dehydrogenase (DHODH) dehydrogenizes dihydroorotate to orotic acid that is the key step of biosynthesis of de novo pyrimidine to generate uracil essential for replication of both viral RNA and DNA (Evans and Guy, 2004). Compound A3, which was identified by high-throughput screening of 61,600 small-molecular-weight compounds, is able to target DHODH, interrupt the biosynthesis of pyrimidine, and inhibit replication of viruses including negative strand RNA viruses (such as IFV-A and B), positive strand RNA viruses (such as hepatitis C virus, HCV and DENV), and DNA viruses (such as VACV and adenovirus; Hoffmann et al., 2011). Compound A3 treatment also causes damages to viral genomic RNA and reduces viral infectivity (Ortiz-Riano et al., 2014). Co-treatment with Ribavirin enhances the therapeutic efficacy of A3 in control of LCMV (Ortiz-Riano et al., 2014). Ribavirin is a broad-spectrum antiviral agent and is approved for treatment of RSV and HCV infection (Naik and Tyagi, 2012). Ribavirin, a guanosine analog, has been used to inhibit synthesis of viral RNAs (Parker, 2005). Ribavirin also inhibits RNA polymerase of HCV to interfere with viral replication and induces formation of GTP analog RTP to cause translational error of viral proteins (Cannon et al., 2009; Shah et al., 2010). In addition, Ribavirin is known to inhibit replication of RNA and DNA viruses via distinctive mechanisms and replication of the same virus via distinct mechanisms in different cell types, depending on intracellular Ribavirin metabolism (Shah et al., 2010). Interestingly, treatment with Ribavirin alone showed a modest or no effect on viral replication in HCV-infected patients; however, combination with IFN dramatically improved long-term antiviral response in patients (Dixit and Perelson, 2006). Another DHODH inhibitor, compound 1 (Cmp1), was identified by the silico fragment-based drug design (FBDD); Cmp1 inhibits both human and murine DHODH, *in vitro,* in a dose-dependent manner (Konteatis, 2010). Cmp1, at low micro- to nano-molar levels, is highly effective in inhibiting replication of human and murine cytomegaloviruses (HCMV and MCMV) as well as HAdV-2 (Marschall et al., 2013). An *in vivo* study showed that treatment of mice with Cmp1 at 30 mg/kg/day for 7 days resulted in a significant suppression of MCMV replication in various organs, including salivary gland, lung, liver, and spleen (Marschall et al., 2013).

Antimycin A, isolated from *Streptomyces kaviengensis*, displays a potent activity to control equine encephalitis viruses (EEV) infection; treatment with antimycin A increased survival rates of EEV-infected mice and reduced viral replication (Raveh et al., 2013). Antimycin A is also able to control infection by various RNA viruses, including members of the *Togaviridae*, *Flaviviridae*, *Bunyaviridae*, *Picornaviridae*, and *Paramyxoviridae* families (Raveh et al., 2013). Interestingly, antimycin A inhibits cellular mitochondrial electron transport chain and de novo pyrimidine synthesis, indicating that host mitochondrial electron transport is a viable target for identifying broad-spectrum antiviral agents. The commercialized antiprotozoal agent Nitazoxanide has been recently identified as a first-in-class broad-spectrum antiviral agent to inhibit the replication of various RNA and DNA viruses, including HIV, IFV, DENV, HCV, HBV, and JEV (Rossignol, 2014). Nitazoxanide is shown to mobilize Ca^2+^, resulting in chronic sub-lethal stress of ER and disruption of viral protein glycosylation and trafficking (Ashiru et al., 2014).

Based on phylogenetic analysis, positive-sense RNA viruses picornaviruses, caliciviruses, and coronaviruses are recognized as members of picornavirus-like supercluster. These viruses possess 3C or 3C-like proteases (3Cpro or 3CLpro, respectively), which contain a typical chymotrypsin-like fold and a catalytic triad (or dyad) with a Cys residue as a nucleophile (Xia and Kang, 2011). 3Cpro and 3CLpro act as key enzymes for proteolytic processing of viral polyproteins for virus replication (Wang and Liang, 2010). To target 3Cpro and 3CLpro of supercluster members, the aldehyde GC373, the α-ketoamide GC375, and the bisulfite adduct GC376, which share a common dipeptidyl residue, are identified with the ability to inhibit the enzymatic activity of 3Cpro or 3CLpro via covalent binding to a nucleophilic Cys residue in the conserved catalytic site of 3Cpro and 3CLpro (Kim et al., 2012, 2013). GC373, GC375, and GC376 are being developed as broad-spectrum antiviral agents to control viral infection by picornavirus-like supercluster members.

### Viral Assembly and Budding

Formation of viral ribonucleoprotein (vRNP) complexes, consisting of viral RNA, RNA polymerase, and nucleoprotein, is essential for the process in replication of negative-sense single-stranded RNA viruses, such as IFV-A. After replication of IFV genomic RNA in the nucleus, the vRNP complexes translocate into cytoplasm for assembly of viral nucleocapsid through a series of nuclear export pathways, such as the nuclear export receptor chromosome region maintenance 1 (CRM1)-mediated pathway (Mao and Yang, 2013). Leptomycin B (LMB) is a highly specific inhibitor of CRM1, and LMB treatment blocks the export of vRNP from nucleus to cytoplasm, thereby suppressing the assembly of IFV-B particles (Dauber et al., 2009). LMB has also been shown to inhibit replications of DNA viruses, such as HSV-1 (Park et al., 2013). Although LMB treatment was effective, its toxicity was detectable in animals (Perwitasari et al., 2014). Thus, LMB needs to be further studied and modified to improve its therapeutic value in control of viral infection.

The human CRM1 homolog exportin 1 (XPO1) mediates transport of leucine-rich nuclear export signal-dependent cellular protein and RNAs for cell growth and differentiation (Askjaer et al., 1998). The nuclear export of vRNPs complexes is mediated by the interaction of XPO1 with viral nuclear export protein of IFV-A NS2 (Neumann et al., 2000). KPT-335 (also Verdinexor) is a small molecule antagonist of XPO1 (Perwitasari et al., 2014). *In vitro* studies showed that KPT-335 is able to selectively and effectively inhibit replication of various IFV A and B strains, including pandemic H1N1, highly pathogenic AIV H5N1, and the recently emerged H7N9. *In vivo* studies showed that prophylactic and therapeutic administration of KPT-335 protected mice from IFV A/California/04/09 or IFV A/Philippines/2/82-X79, and KPT-335 treatment reduced production of viral particles and proinflammatory cytokines in lung with non-significant toxicity (Perwitasari et al., 2014). Thus, KPT-335 appears to be a promising broad-spectrum antiviral agent.

It has been recently reported that enveloped viruses, such as EBOV, utilize the cellular endosomal sorting complexes required for transport (ESCRT) pathway to release their progenies from infected cells; the ESCRT pathway is also the major escaping route for enveloped viruses (Votteler and Schubert, 2008; Bucci, 2013; Snyder et al., 2013; Dolnik et al., 2014). The tumor susceptibility gene 101 product TSG101 is a subunit of ESCRT-I (Luyet et al., 2008). The phenolic compound FGI-104 targets TSG101 and inhibits viral budding of EBOV; treatment with FGI-104 at 10 mg/kg/day for 7 days protected mice from EBOV-caused death. FGI-104 treatment also intervenes in HCV, HIV-1, IFV-A, and HSV-1 infection via inhibition of TSG101 (Kinch et al., 2009). Recently, Lu’s group reported that TSG101 serves as a target of the Z protein PTAP-L domain of JUNV, which causes Argentine hemorrhagic fever, to facilitate viral budding (Lu et al., 2014). Using the structure of TSG101-PTAP interaction site as a target for silico screening of competitive binding molecules, a novel compound 0013 was identified by its ability to block the interaction of protein Z with TSG101 (Lu et al., 2014); the 0013 inhibited budding of JUNV particles. It is known that PTAP L-domain-containing proteins are generally required for separation of RNA viral particles from host cells (Chen and Lamb, 2008). Studies showed that the 0013 is effective in inhibiting PTAP-dependent budding of arenavirus, filovirus, and retrovirus (Lu et al., 2014). Thus, compound 0013 can be used as a lead compound for further development of broad-spectrum antiviral agents to control viral budding.

### Viral Envelope

Cells are able to promptly repair damages, ranging from 0.2 to 10 μm, of cell membrane by cytoplasmic endomembrane; however, viral particles are incapable of repairing damaged viral envelope (McMahon and Gallop, 2005). LJ001 is a type II photosensitizer derived from aromatic methyl diene rhodanine (Wolf et al., 2010). Treatment of cells with LJ001 results in generating singlet oxygen (^1^O_2_) in the membrane bilayer, and ^1^O_2_-mediated lipid oxidation permanently changes biophysical properties of viral envelope, resulting in interruption of virus-cell fusion; in contrast, LJ001 treatment does not result in a permanent damage of cell membrane due to the ability of cells to repair lipid biosynthesis (Wolf et al., 2010). Thus, LJ001 treatment is able to irreversibly damage the viral envelope bilayer structure, but not host cell membrane, to protect cells from fusion with enveloped viruses, including bunyaviruses, filoviruses, poxviruses, arenaviruses, paramyxoviruses, flaviviruses, IFV-A, and HIV-1 *in vitro* (Wolf et al., 2010). Using LJ001 as a lead compound, a new class of membrane-targeted photosensitizers (i.e., oxazolidine-2,4-dithione derivatives: JL118 and JL122) has been developed to improve solubility and bioavailability for *in vivo* application. *In vitro* studies showed that JL118 and JL122 are able to increase ^1^O_2_ level 100-fold than LJ001 can do in cells. *In vivo* studies showed that JL118 and JL122 treatment significantly delayed the death of RVFV (bunyaviruses)-infected mice (Vigant et al., 2013).

Recently, mastoparan, which is a natural host defense peptide (NHP) isolated from the invertebrate wasp, has been shown to enter into viral envelop and interact with the lipid components (Albiol Matanic and Castilla, 2004). The mastoparan-derived peptide MP7-NH2 is able to alter the structure of viral envelope and directly inactivate the infectivity of enveloped viruses, including rhabdoviruses, flaviviruses, herpesviruses, and poxviruses, but not IFV-A (Sample et al., 2013). Possibly, IFV-A envelope is less organized/ordered than the envelope of viruses, such as VSV of rhabdoviruses, contributing to IFV resistance to MP7-NH2 (Doms et al., 1988; Mukhopadhyay et al., 2005; Harris et al., 2006). Pre-exposure of VSV particles to MP7-NH2 significantly reduced the infectivity of VSV *in vivo* (Sample et al., 2013). Thus, LJ001 and MP7-NH2 should be recognized as lead compounds for further development of broad-spectrum agents to control viral infection by targeting viral envelope.

The indole derivative Arbidol has been shown to block the interaction between cell membrane and HCV glycoproteins to inhibit viral entry and fusion into cells (Boriskin et al., 2008). Arbidol also inhibits viral replication of HCV by altering cytoplasmic proteins, which are essential for intracellular trafficking (e.g., clathrin coat components, elements of the cytoskeleton) and viral replication (e.g., membranous web), and by hindering membrane rearrangements involved in viral budding. Arbidol has been clinically shown to inhibit infection by enveloped viruses, such as IFV-A and coronavirus, as well as non-enveloped reovirus; thus, Arbidol is considered as a broad-spectrum antiviral agent worthy of further development (Blaising et al., 2014). Arbidol has been clinically used to treat viral infections in Russia since 2005; however, it has not been approved for use in Western countries (Blaising et al., 2014).

### Summary for the First Section

In general, antiviral agents, such as DAS181 and C-AU, which target viral attachment or virus-cell membrane fusion/endocytosis are specific to viruses; however, their treatments often result in drug-resistant viral variants, and their specificity to viruses limits their potential for development of broad-spectrum antiviral agents. Agents, such as BCX4430 and JMN3-003, inhibit viral RNA polymerases and are non-cytotoxic; their ability to block replication of various RNA viruses without resulting in drug-resistant viral variants positions their candidacy to be developed into optimal broad-spectrum agents to inhibit RNA viruses. Compound A3, which interrupts biosynthesis of pyrimidine in cells, appears to be a broad-spectrum antiviral agent to control both RNA and DNA viruses; A3 treatment does not result in drug-resistant viral variants. KPT-335, which blocks viral assembly in cytoplasm, appears to be another promising broad-spectrum antiviral agent to control RNA and DNA viruses. FGI-104, which targets the cellular exporting ESCRT system, inhibits viral budding without resulting in drug-resistant viral variants. Although compound A3, KPT-335, and FGI-104, which target on cellular machinery, are able to act as broad-spectrum antiviral agents, their potential side effects on patients need to be addressed. Whether combination of these agents will be optimal to control various viruses remains to be determined.

## Agents Based on Host Defense

Host cellular defense is the first hurdle against viral infection. However, viruses are able to utilize cellular machineries for viral replication. Growing knowledge has helped identify broad-spectrum antiviral agents by targeting the cellular machineries of defense, programmed cell death, and metabolism, as presented in the following subsections, and in **Table 2** and **Figure 1**.

**Table 2:** The antivirals based on host defense.

Name	Mechanism/Target position	Derivation	Target viruses	Experiment	Reference
5′pppRNA	RIG-I agonist	From viral RNA structures	IFV-A and VSV, etc	*In vivo*	Goulet et al. (2013)
ISG15	Distinctive mechanisms	Knockdown	RNA viruses, e.g., HIV-1 and IFV-A	*In vivo*	Morales and Lenschow (2013) (Review)
ITIF	Distinctive mechanisms	Knockdown	Broad-spectrum, e.g., HPV and IFV-A	*In vivo*	Diamond and Farzan (2013) (Review)
IFITM	Distinctive mechanisms	Knockdown	Enveloped viruses, e.g., IFV-A and SARS-CoV	*In vivo*	Diamond and Farzan (2013) (Review)
C1 and C2	Rnase L activator	Synthesis	RNA viruses, e.g., PIV and VSV	*In vitro*	Thakur et al. (2007)
GSK983	Induce ISGs	Synthesis	HPV and EBV, etc	*In vivo*	Harvey et al. (2009)
Akt-IV	P13k/Akt inhibitor	Synthesis	VSV and RSV, etc	*In vitro*	Dunn et al. (2009)
BEZ-235	PI3K inhibitor	Synthesis	LCMV and JUNV, etc	*In vitro*	Urata et al. (2012)
DRACOs	Induce apoptosis	Recombinant	Broad-spectrum, e.g., IFV-A	*In vivo*	Rider et al. (2011)
Rapamycin	mTOR pathway inhibitor	Extract from *Streptomyces*	Retroviruses, e.g., HIV-1	*In vivo*	Nicoletti et al. (2011)
Baicalin	Attenuate autophagy	Extract from *Scutellariaradix*	IFV-A and DENV, etc	*In vivo*	Zhu et al. (2015)
MG132	Proteasome inhibitor	Synthesis	Broad-spectrum, e.g., HIV-1 and IFV-A	*In vitro*	Schubert et al. (2000)
Bortezomib	Proteasome inhibitor	Synthesis	Broad-spectrum, e.g., IFV-A and VSV	*In vitro*	Dudek et al. (2010)
VL-01	Proteasome inhibitor	Synthesis	A serotypes of IFV-A	*In vivo*	Haasbach et al. (2011)
Mucin polymer	Physical barrier from viruses	Gastric mucus extracts	Broad-spectrum, e.g., HPV and IFV-A	*In vitro*	Lieleg et al. (2012)

### Interferon (IFNs)

Type I IFNs [alpha and beta interferons (IFN-α/β)] are the first-line cytokines for host’s antiviral defense via immune system and rapid induction of cellular modulators to inhibit viral replication and spread (Burke et al., 2014). Viral infection yields highly conserved pathogen-associated molecular patterns (PAMPs), such as double-stranded RNA (dsRNA). Viral RNA contains short hairpin dsRNA with triphosphorylated 5′ end (5′pppRNA) that preferentially activates host’s pattern recognition receptors (PRRs), such as retinoic acid inducible gene-I (RIG-I) and related receptors (Ng and Gommerman, 2013). 5′pppRNA-activated RIG-I interacts with mitochondrial antiviral-signaling protein (MAVS) to result in activation of transcription factors, such as IFN regulatory factor-3/7 (IRF-3/7), through the IKK-related kinases TBK1 and IKK𝜀, to induce IFN gene expression (Schlee et al., 2009; Wilkins and Gale, 2010). An analog of 5′pppRNA, derived from the 5′ and 3′ untranslated regions of VSV genome (Schlee et al., 2009), has been shown to induce IFN expression and multiple innate antiviral responses, including IRF3/7, to control infections with IFV-A, DENV, VSV, HCV, and VACV (Goulet et al., 2013; Olagnier et al., 2014). Mice, treated with 5′pppRNA, fully recovered (100% survival) from a lethal infection with H1N1 A/PR/8/34 IFV; 5′pppRNA treatment completely inhibited viral replication in the lung (Goulet et al., 2013).

Secretion of type I IFNs from virus-infected cells may trigger antiviral activity in not only infected cells but also surrounding uninfected cells in an autocrine- or a paracrine-dependent manner. IFNs bind to cell surface IFN receptor (IFNAR) to activate the Janus kinase signal transducer and activator of transcription (JAK-STAT) pathway, leading to upregulation of hundreds of IFN-stimulated gene (ISG) products, such as ISG15, ITIT, IFITM and 2–5′ oligoadenylate synthetase (2-5A OAS; Burke et al., 2014). In general, ISGs have been shown to exhibit a wide range of antiviral abilities, including the ability to degrade viral nucleic acids, to inhibit viral gene expression, and to serve as PRRs to amplify IFN signals and elevate IFN expression (Bisbal et al., 2001; Wilkins and Gale, 2010).

ISG15 may act as an ubiquitin cross-reactive protein (Morales and Lenschow, 2013). ISG15 is able to inhibit ubiquitination of HIV-1 Gag protein and the interaction of Gag with TSG101, which are important for releasing HIV-1 particles from cells (Pincetic and Leis, 2009). ISG15 may block the ESCRT pathway (see *Viral Assembly and Budding*) and disrupt cell release of viral particles, such as HIV-1, EBOV, and ASLV (Morales and Lenschow, 2013). ISG15 is also involved in ISGylation of viral proteins that contributes to IFN-mediated inhibition of virus replication. HERC5 is an IFN-induced E3 ubiquitin ligase for ISG15 to mediate ISGylation of the IFV-A protein NS1 that directly inhibits viral replication (Tang et al., 2010; Zhao et al., 2010). ISG15 is also able to help other ISGs, such as IRF3. ISGylation of IRF3 inhibits ubiquitination and degradation of IRF3 (Lu et al., 2006). Stabilized IRF3 forms complex with CREB binding protein (CBP) that translocates into the nucleus and activates the transcription of IFNs and other ISGs genes (Prinarakis et al., 2008). IRF3 has been shown to play a critical role in the innate immune response against DNA and RNA viruses. Overexpression of ISG15 resulted in increased antiviral activity *in vitro* and *in vivo*, and knockdown of ISG15 resulted in increased replication of VSV, SeV, and NDV in cells (Shi et al., 2010) that further demonstrated the ISG15 antiviral ability.

IFIT (Interferon induced proteins with tetratricopeptide repeats) and IFITM (IFN-induced transmembrane protein) are two families, involved in IFN activation, with distinctive genetics and functions. Both IFIT and IFITM show broad-spectrum antiviral activity to interfere with viral replication, transmission, and virulence (Diamond and Farzan, 2013). Four human family members (IFIT1, IFIT2, IFIT3, and IFIT5) and three rodent family members (Ifit1, Ifit2, Ifit3) are identified. IFIT inhibits viral infections through multiple mechanisms; IFIT1 and IFIT2 suppress the translation initiation (Hui et al., 2003), IFIT1 binds uncapped or incompletely capped viral RNA (Pichlmair et al., 2011) and sequesters viral protein (e.g., HPV E1) or RNA in the cytoplasm (Terenzi et al., 2008). On the other hand, human IFITM family composes of four members IFITM1, IFITM2, IFITM3, and IFITM5; and murine IFITM composes of Ifitm1, Ifitm2, Ifitm3, and Ifitm5. IFITM proteins traverse cell membrane and are abundant in endosomes and lysosomes, and they can prevent enveloped viruses from crossing endosomal or lysosomal membranes and penetrating into the cytoplasm (Diamond and Farzan, 2013). IFITM members also interfere with the membrane fusion and endocytosis of viral particles to effectively inhibit viral infection by enveloped viruses; such as IFV, EBOV, SARS-CoV and DENV (Brass et al., 2009; Huang et al., 2011). It is shown that IFITM3 is able to limit IFV-A to the hemagglutinin attachment phase of viral replication, contributing to control of IFV-A infection *in vivo* (Brass et al., 2009; Feeley et al., 2011). However, the detailed mechanisms for IFITM members in differential inhibition of enveloped viruses and whether IFITM may interfere with replication of non-enveloped viruses remain to be studied.

The 2-5A OAS/RNase L pathway has been detected in various types of mammalian cells (Bisbal et al., 2001). Upon activation by viral dsRNA, 2-5A OAS synthesizes 2–5′ oligoadenylates (2-5A) from ATPs. The resulting 2-5A binds to the inactive monomeric form of RNase L to result in dimerization and activation of RNase L, and activated RNase L mediates degradation of viral RNA, contributing to IFN antiviral activity (Chakrabarti et al., 2011; Silverman and Weiss, 2014). Thus, activators of RNase L are considered for developing antiviral agents. However, 2-5A is labile and cytotoxic; it may not be an optimal antiviral agent candidate (Gale and Sen, 2009). High throughput screening of the chemical library ChemBridge DIVERset of 30,000 small molecules with the technique of fluorescence resonance energy transfer (FRET) has identified seven compounds effective in activating RNase L at micromolar concentrations (Thakur et al., 2007). Two lead compounds, C1 and C2, are able to enter into cells and induce RNase L activity without showing cytotoxicity. Both C1 and C2 show broad-spectrum antiviral activity against RNA viruses, including picornaviruses, rhabdoviruses, paramyxoviruses, and retroviruses (Thakur et al., 2007). In addition, GSK983, a novel tetrahydrocarbazole, has been shown to induce significant increases of seven ISGs, including ISG15, ISG56, OAS1, OAS2, OASL, IL6, and TRAIL. GSK983 treatment resulted in growth inhibition of cells infected by HPV, adenovirus Ad-5, or EBV, possibly via induction of ISGs (Harvey et al., 2009). However, the exact mechanism for GSK983 antiviral activity still needs a clarification.

### Programmed Cell Death

Apoptosis and autophagy are two major forms of programmed cell death and play essential roles in not only homeostasis but also survival in multicellular organisms (Lockshin and Zakeri, 2007; He and Klionsky, 2009). Apoptosis and autophagy are distinguished on the basis of cell morphological changes (Hacker, 2000; Lockshin and Zakeri, 2007). Apoptosis is characterized by activation of proteolytic caspases and formation of apoptotic bodies that mediate degradation of cellular organelles. Autophagy is characterized by the formation of autophagosomes to sequestrate and transport cytoplasmic materials, including damaged proteins and organelles, to lysosomes for degradation; it is regarded as “self-eating” catabolism.

In response to viral infection, induction of apoptosis may limit viral production and spread into adjacent cells, while simultaneously promoting host innate and inflammatory responses (Taylor et al., 2008; Upton and Chan, 2014). However, viruses, such as HSV-1 and EBOV, are able to escape from apoptotic control or reverse the process to facilitate viral replication, respectively (Martins et al., 2012; Fuentes-Gonzalez et al., 2013). Infection of viruses, such as paramyxoviruses and orthomyxoviruses, may result in activation of the phosphatidylinositol 3-kinase (PI3K)/Akt pathway to block apoptosis for the benefit of viral replications (Sun et al., 2008; Liu et al., 2010; Diehl and Schaal, 2013). Activated Akt phosphorylates downstream transcription factors, such as FoxO1; FoxO1 promotes degradation of IRF3 in suppression of cellular antiviral response (see *Programmed Cell Death*; Lei et al., 2013). The PI3K/Akt pathway inhibitor Akt-IV was identified by high throughput screening of inhibitors of FoxO1 (Ramaswamy et al., 1999; Sun et al., 2008). Akt-IV has been shown to inhibit replication of the paramyxoviruses PIV5 and RSV. Interestingly, although the PI3k/Akt pathway is not involved in the replication of VSV, Akt-IV is able to inhibit VSV replication (Sun et al., 2008; Dunn et al., 2009). It has also been shown that the PI3K inhibitor BEZ-235 is effective in inhibiting the production of LCMV and JUNV viral particles without causing cell toxicity (Urata et al., 2012).

The dsRNA-activated caspase oligomerizer (DRACO) is a new antiviral agent and is currently under preclinical studies. DRACO consists of three components: a dsRNA recognition domain derived from protein kinase R (PKR) for activating the defensive IFN pathway, apoptotic protease activating factor-1 (Apaf-1) for inducing apoptotic caspase pathways, and a transduction peptide derived from HIV-1 TAT protein for entering into cells (Rider et al., 2011). Thus, the DRACO approach induces both IFN and apoptosis pathways for controlling virus-infected cells. Studies showed that DRACO is able to induce death of cells infected by either enveloped or non-enveloped viruses with undetectable toxicity to uninfected cells (Rider et al., 2011). DRACO has been reportedly to effectively control infections by various viruses, including orthomyxovirus, flavivirus, picornavirus, arenavirus, bunyavirus, reovirus, and adenovirus (Rider et al., 2011). Animal studies showed that administration of DRACO from 1 day prior to viral infection through 3 days post to viral infection significantly reduced production of viral particles and morbidity of animals intranasally infected with IFV H1N1 A/PR/8/34 (Rider et al., 2011). DRACO appears to be an optimal broad-spectrum antiviral agent for further development.

Autophagy is a basic cellular mechanism for degradation of unnecessary or dysfunctional cellular components through the actions of lysosomes to recycle cellular components (He and Klionsky, 2009). Autophagy is reportedly involved in both pro-survival and pro-death pathways depending on individual cellular events (Nikoletopoulou et al., 2013). Autophagy has been recognized to play roles in controlling intracellular pathogens and fueling innate and adaptive immune responses to viral infections (Liang et al., 1998; Nakashima et al., 2006; He and Klionsky, 2009). Viruses, such as HSV-1 ICP34.5, can block the formation of autolysosomes by targeting the autophagic protein Beclin-1 to inhibit autophagy and allow viral replication (Orvedahl et al., 2007). The non-structural protein 2C of foot-and-mouth disease virus (FMDV) can interact with Beclin-1 for facilitating viral replication (Gladue et al., 2012). Viruses, such as flaviviruses, orthomyxoviruses, and coronaviruses, may also utilize autophagic machinery to facilitate viral replication (Prentice et al., 2004; Jackson et al., 2005; Zhou et al., 2009; Heaton and Randall, 2011; Dumit and Dengjel, 2012). Thus, whether autophagy is an optimal machinery targeted for developing broad-spectrum antiviral agents remains to be addressed. Treatment with the autophagy inhibitor Baicalin, a natural product isolated from *Scutellariaradix*, resulted in suppressing expression of autophagic Atg5–Atg12 complex and autophagosomal marker LC3-II in cells (Zhu et al., 2015). Baicalin treatment has been shown to suppress viral replication of the flavivirus DENV *in vitro* (Moghaddam et al., 2014). Baicalin treatment also attenuated IFV-A-induced autophagy in cells and effectively increased survival rates of mice infected with IFV H3N2 or H1N1 (Nayak et al., 2014; Zhu et al., 2015).

Mammalian target of rapamycin (mTOR) has been shown to play roles in regulation of cell growth and autophagy in response to changes of nutritional supplies, growth factors, and stresses (Jung et al., 2010). The mTOR is a serine/threonine kinase and mediates signals to block autophagy via phosphorylation of Atg13 and ULK1/2. Treatment of cells with the mTOR inhibitor Rapamycin leads to dephosphorylation of ULK1/2 and Atg13, resulting in induction of autophagy (Jung et al., 2010). HIV-1 infection may result in induction of mTOR for protein synthesis that downregulates autophagy in lymphocytes and dendritic cells, contributing to its blockage of host’s immune responses (Zhou and Spector, 2008; Gougeon and Piacentini, 2009; van der Vlist et al., 2011; Rehman et al., 2012). Treatment with Rapamycin blocked HIV-1 infection of T cells *in vitro* (Roy et al., 2002; Oswald-Richter et al., 2004) and inhibited HIV-1 infection of human leucocytes in SCID mice (Nicoletti et al., 2009; Nicoletti et al., 2011). Combination of Rapamycin with the viral entry inhibitor Vicriviroc synergistically enhanced suppression of HIV-1 infection *in vitro* (Heredia et al., 2008). Rapamycin treatment has also been shown to reduce replication of avian leukemia virus (ALV; Liu et al., 2013). These results suggested that Rapamycin is a potential candidate for anti-HIV drug development. However, how to adequately use agents to suppress or activate autophagy for control of viral infection still needs to be addressed.

### Ubiquitin-Proteasome System (UPS)

Ubiquitination and ubiquitination-like modifications (e.g., SUMOylation) are widely involved in stability, localization, and recycling of cellular proteins, as well as interactions of proteins with their substrates (Hershko, 1983; Hochstrasser, 2009). Ubiquitination and ubiquitination-like modifications play roles in regulation of cellular proteins for nuclear transportation, proteolysis, translation, autophagy, and antiviral responses. Ubiquitination is a post-translational modification via attachment of ubiquitin (UB) to a substrate protein. UB is a small regulatory protein (MW 8.5 kDa) and found in almost all eukaryotic cells. Ubiquitination is initially induced by interaction of the UB-activating enzyme E1 to activate UB, followed by interaction with the UB-conjugating enzyme E2 and the UB ligase E3 to form an isopeptide bond between the carboxyl terminus of UB and the 𝜀-amino group of lysine residues on a targeted protein (Pickart, 2001). Subsequently, the resulting UB-tagged protein binds to proteasome for degradation and recycling. Accordingly, assembly of ubiquitination-involved components and proteasomes together constructs the UB-proteasome system (UPS; Coux et al., 1996).

Ubiquitination and ubiquitination-like modifications are known to play important roles in regulation of viral components, such as proteasomal degradation of HBV X protein (Kim et al., 2008), HCV core protein (Shirakura et al., 2007), and HAV 3C proteases (Schlax et al., 2007). It is logical to enhance the host cell defense ability, via modulation of ubiquitin system, to control viral infection. However, it has been reported that viruses are able to utilize UPS to help viral replication (Isaacson and Ploegh, 2009). For example, HIV-1 and IFV-A and B utilize UPS to modify their viral proteins HIV-Gag and IFV-M1, respectively, to facilitate viral replication. Ubiquitination of HIV-Gag, which is a major structural protein, enhances its binding to TSG101 (subunit of ESCRT-I, see *Viral Assembly and Budding*) for assembly of viral particles and completion of viral budding and release (Pornillos et al., 2002; Klinger and Schubert, 2005). SUMOylation of IFV-A-M1 plays an important role in modulating assembly of viral particles (Pal et al., 2011; Rossman and Lamb, 2011; Wu et al., 2011). Viruses are also able to utilize UPS to subvert or interfere with host innate defense system (such as APOBEC3G and NF-kB). The HIV-1 proteins Vif, Nef, Vpu, and Vpr, are essential for viral replication, and they are important for evading host immune system (Strebel, 2013). For example, the unique zinc-finger motif of Vif recruits E3 ubiquitin ligase to form a complex of CBF-β-CUL5-ELOB-ELOC, which ubiquitinates the human antiviral protein APOBEC3G for degradation (Huthoff and Towers, 2008; Guo et al., 2014). NF-kB is sequestered by IkB in the cytoplasm; viral infection, such as HIV-1 and EBV, may result in the activation of NF-kB through UPS-dependent degradation of IkB, leading to up-regulation of a variety of antiviral genes (Magnani et al., 2000; Mingyan et al., 2009; Sun and Cesarman, 2011). However, IFV-A is able to utilize NF-kB-dependent signaling pathways, via degradation of IkB to activate NF-kB, for viral replication (Ludwig and Planz, 2008). It has been reported that the proteasome inhibitors MG-132 (Carbobenzoxy-L-leucyl-L-leucyl-L-leucinal, peptide aldehyde), Lactacystin, and Bortezomib are able to block NF-kB activation, via inhibition of IkB degradation (Omura et al., 1991; Palombella et al., 1994; Hideshima et al., 2009). MG132 treatment inhibits IFV-A replication (Widjaja et al., 2010). Treatment of cells with Bortezomib (PS-341), at non-cytotoxic levels, significantly reduces IFV-A and VSV replication (Dudek et al., 2010). The proteasome inhibitor VL-01 is able reduce viral replication of three IFV strains, including A/Puerto Rico/8/34 (H1N1), A/Regensburg/D6/09 (H1N1v) and A/Mallard/Bavaria/1/2006 (H5N1). Treatment of IFV-infected mice with VL-01 resulted in reducing viral replication in the lung and increasing mice survival without any side effects (Haasbach et al., 2011).

Paramyxoviruses (SeV and RSV) may induce ubiquitination of STATs to evade host IFN response [see *Interferon (IFNs)*]; the SeV C protein and the RSV NS protein are able to induce ubiquitination of STAT1 and STAT2, respectively, for degradation (Garcin et al., 2002; Elliott et al., 2007). Treatment of SeV- or RSV-infected cells with MG132 reduced viral replication in a dose-dependent manner (Watanabe et al., 2005; Lupfer and Pastey, 2010). More than ten families of viruses utilize UPS to complete their replication for a successful infection (Calistri et al., 2014). Treatment with MG-132 or Lactacystin effectively blocks maturation, budding, and infectivity of HIV-1 particles (Schubert et al., 2000; Klinger and Schubert, 2005; Votteler and Schubert, 2008). Proteasome inhibitors have also been shown to interfere with replication of herpesviruses (Delboy et al., 2008), coronaviruses (Yu and Lai, 2005), and rotaviruses (López et al., 2011). Thus, it is promising to develop optimal broad-spectrum non-cytotoxic antiviral agents by inhibiting UPS.

### Native Barrier

Mucus plays an important role in protection of epithelial cells from invading pathogens in the respiratory, gastrointestinal, urogenital, visual, and auditory systems of animals (Thornton and Sheehan, 2004; Martinez et al., 2015). Mucins, the major components of mucus, are a family of high molecular weight glycoproteins (up to hundreds of kDa) produced by epithelial tissues in both vertebrates and invertebrates. Mucins are able to form a gel-like barrier to protect cells from viral attachment (Lieleg et al., 2012). Porcine mucins, isolated from gastric mucous epithelium, has been shown to form a shield to trap viral particles, such as HPV and IFV IFV-A with sizes of ∼50 and∼100 nm, respectively (Buck et al., 2005), as well as HIV (∼120 nm) and HSV (∼180 nm; Lai et al., 2010; Lieleg et al., 2012). Porcine gastric mucins may be used as antiviral agents for future biomedical applications.

### Summary for the Second Section

More than 200 cellular functional proteins are directly and indirectly involved in replication of HIV-1 (Brass et al., 2008; Dziuba et al., 2012). Understanding the roles of cellular proteins, and associated pathways, in viral replication has helped design new strategies for developing broad-spectrum antiviral agents, such as the PI3K inhibitor Akt-IV and BEZ-235, the mTOR inhibitor Rapamycin, and the proteasome inhibitors MG132 and VL-01. Eventually, each agent has its own specificity to intervene in replication of targeted viruses. However, challenges of drug toxicity to hosts and generation of resistant viral progenies remain to be addressed.

## Conclusion and Perspectives

Current development of broad-spectrum antiviral agents targeting viral infectivity and modulating host defense system has substantially advanced the fields of virology and pharmaceutics and significantly contributed to the health care of human and animals. However, there are concerns of viral resistance associated with agents targeting viral components and non-specific side effects associated with agents targeting cellular machineries. Accordingly, how to reduce viral resistance and increase drug specificity are current challenges to be addressed. Whether combined uses of agents to target both viral components and cellular machineries may improve antiviral efficacy, reduce viral resistance, and minimize toxicity in the control of viral infection and epidemic viral diseases needs to be clarified.

## Conflict of Interest Statement

The authors declare that the research was conducted in the absence of any commercial or financial relationships that could be construed as a potential conflict of interest.

## Abbreviations

AIV: avian influenza virus
ASLV: avian sarcoma leucosis virus
DENV: dengue virus
EBOV: Ebola virus
EBV: Epstein-Barr virus
HAdV-2: human adenovirus type 2
HBV: hepatitis B virus
HCMV: human cytomegalo virus
HCV: hepatitis C virus
HeV: Hendra virus
HIV: human immunodeficiency virus
HPV: human papillomavirus
HSV-1: herpes simplex virus 1
IBV: infectious bronchitis virus
IFV-A: influenza virus Type A
IFV-B: influenza virus Type B
JEV: Japanese encephalitis virus
JUNV: Junin arenavirus
LCMV: lymphocytic choriomeningitis virus
LFV: Lassa fever virus
MCMV: murine cytomegalo virus
NDV: Newcastle disease virus
NiV: Nipah virus
PIV: parainfluenza virus
RSV: respiratory syncytial virus
RVFV: Rift Valley fever virus
SARS-CoV: severe acute respiratory syndrome coronavirus
SeV: sendai virus
VACV: vaccinia virus
VSV: vesicular stomatitis virus
WEEV: equine encephalitis virus
YFV: yellow fever virus.
